# DenseTextPVT: Pyramid Vision Transformer with Deep Multi-Scale Feature Refinement Network for Dense Text Detection

**DOI:** 10.3390/s23135889

**Published:** 2023-06-25

**Authors:** My-Tham Dinh, Deok-Jai Choi, Guee-Sang Lee

**Affiliations:** Department of Artificial Intelligence Convergence, Chonnam National University, 77 Yongbong-ro, Gwangju 500-757, Republic of Korea; thamdinh.dmt@gmail.com (M.-T.D.);

**Keywords:** scene text detection, pyramid vision transformer, dense adjacent text

## Abstract

Detecting dense text in scene images is a challenging task due to the high variability, complexity, and overlapping of text areas. To adequately distinguish text instances with high density in scenes, we propose an efficient approach called DenseTextPVT. We first generated high-resolution features at different levels to enable accurate dense text detection, which is essential for dense prediction tasks. Additionally, to enhance the feature representation, we designed the Deep Multi-scale Feature Refinement Network (DMFRN), which effectively detects texts of varying sizes, shapes, and fonts, including small-scale texts. DenseTextPVT, then, is inspired by Pixel Aggregation (PA) similarity vector algorithms to cluster text pixels into correct text kernels in the post-processing step. In this way, our proposed method enhances the precision of text detection and effectively reduces overlapping between text regions under dense adjacent text in natural images. The comprehensive experiments indicate the effectiveness of our method on the TotalText, CTW1500, and ICDAR-2015 benchmark datasets in comparison to existing methods.

## 1. Introduction

Scene text detection has made significant progress in computer vision and plays a crucial role in various practical applications such as scene understanding, scene reading, and autonomous driving. The application of deep learning has led to remarkable achievements in detecting text in natural scenes [1,2,3,4,5,6,7,8,9,10,11,12,13,14,15].

**Figure 1:**
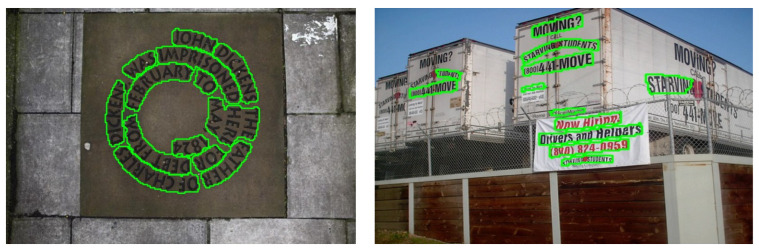
Sample of inaccurate dense predictions in previous works.

Recent methods in scene text detection have extensively utilized deep neural networks (DNNs) to extract features and achieve impressive performance on benchmark datasets [16,17,18]. Despite these advancements, scene text detection remains a challenging task, primarily due to the irregular shapes, diverse scales, and high density of text instances in scenes (as illustrated in Figure 1). Existing methods like SegLink++ [13] and MSR [19] have shown effectiveness in handling text lines and accommodating variations in text line length. However, they have still faced difficulties in dealing with overlapping dense text regions, especially in small-scale texts. Following that, methods like PAN [1], TextSnake [20], and CT [12] aim to address overlap phenomena by expanding text regions from text kernels, but they fall short in achieving competitive results in scene text detection.

To overcome these challenges, our approach explores a multi-scale strategy with three different kernel filters and attention mechanisms, namely, Deep Multi-scale Feature Refinement Network (DMFRN). This method generates and fuses the multi-level features that provide comprehensive representations for scene text instances.

Moreover, this study is inspired by the merits of Transformer [21,22,23,24,25,26], which has been employed to eliminate the complex and understand spatial arrangement and contextual information in manually designed procedures of object detection. Transformer models like DETR [22] tackle the object detection task in a fully end-to-end manner, eliminating the need for complex handcrafted components such as anchor generation, region proposal networks, and non-maximum suppression. However, they are not capable of effectively extracting low-level visual features at a local level effectively, and they also struggle to detect small objects. Although ViT [24] employs a self-attention mechanism within Transformer to model the interactions between patches, enabling the model to capture both local and global contextual information, ViT has struggled to achieve pixel-level dense prediction.

In this work, we propose a solution to accurately predict dense text by employing the PvTv2 versatile backbone [26], which is designed to achieve high output resolution for dense prediction tasks in object detection while reducing resource consumption through a progressive shrinking pyramid. Unlike the original backbone, we added a channel attention module (CAM) and spatial attention module (SAM) between feature levels to effectively capture and leverage informative features in both the channel-wise and spatial dimensions. This work leads to enlarging the receptive fields and preserving high-resolution features, which is crucial for the dense prediction task.

To further enhance the quality of the feature representation, we incorporated a post-processing step based on PAN [1]. This step is designed to reduce the overlap between text regions. By applying this post-processing technique, we can improve the accuracy and clarity of the detected text regions, leading to more reliable results.

Our core contributions are as follows:

1. We propose an effective approach, called DenseTextPVT, which incorporates the advantages of dense prediction backbone in object detection tasks, Pyramid Vision Transformer (PvTv2) [26], with a channel attention module (CAM) [27] and spatial attention module (SAM) [27] to obtain high-resolution features that make our model well suited for dense text prediction in natural scene images.
2. We employed a Deep Multi-scale Feature Refinement Network (DMFRN) using three kernel filters simultaneously (3×3, 5×5, 7×7) with CBAM [27] at each feature. This allows for adaptive feature refinement, enabling our model to enrich feature representations with different scales, including small representations.

The paper consists of the following sections: Section 2 provides a summary of related works in scene text detection and Transformer. Section 3 describes the architecture of the proposed method in detail. Section 4 presents experimental results. Finally, Section 5 concludes the paper and discusses future work.

## 2. Related Work

### 2.1. Scene Text Detection

**The regression-based method** [8,9,10,11,15,28] directly adopts bounding boxes annotation regarding text as an object. He et al. [15] proposed a method for detecting multi-oriented text in scene images using a deep regression network. They utilized semantic segmentation at the pixel level to classify the text and directly calculated offsets between a pixel point and the corresponding box vertices to determine the text quadrangle. SegLink++ [13] presented an approach to detect dense and arbitrarily shaped text in scene images using a network that leverages instance-aware component grouping (ICG). EAST [8] predicted the multi-orientation of text lines or words within the full image directly by employing a fully convolutional network (FCN). FCE [28] formulated text contours in the Fourier domain and represented these arbitrarily shaped texts as compact signatures. Despite their ability to handle text instances with arbitrary shapes, they may struggle with text lines that are challenging to orient and tiny texts.

**The segmentation-based method** [1,2,3,6,7] mainly focuses on pixel-level feature representations [1,2,3,7,29], or segment-level [11,20] or contour-level segmentation [9,30,31]. Typically, these methods usually first segment text kernels and then cluster them into text instances via post-processing. For instance, PSENet [7] utilized a progressive scale algorithm to create a variety of kernels for each text instance and expand, bit by bit, the kernel to cover the entire text instance. Similarly, CT [12] predicted text instances by using text kernels and centripetal shifts, which were used to aggregate pixels, and then directing external text pixels towards the internal text kernels. PAN [1] implemented a clustering approach to precisely aggregate text pixels to exact text kernels based on the similarity vectors. DB++ [2] is an extension of the previous work on differentiable binarization (DB) [29], which incorporated the binarization process into a segmentation network for more accurate results. [32] employed an effective central text region mask and adjusted the expanding ratio from the central text region to the full text instance. However, the performance of these methods is heavily influenced by the quality of the segmentation accuracy.

### 2.2. Transformer

Transformer has become an increasingly popular topic of research in computer vision. Ref. [21] was the accredited father of Transformer, which was based solely on attention modules. Inspired by this architecture, refs. [21,22,23,24,25,26,33,34,35] utilized Transformer-based architecture to approach object detection as a problem of predicting sets. Transformer introduced a simple end-to-end framework that eliminated the need for intricate, hand-crafted anchor generation and post-processing steps. ViT [24] is a Transformer architecture specifically designed for computer vision tasks, and has demonstrated outstanding performance on image classification tasks by directly applying the Transformer to sequences of image patches. DeiT [25] was an extension of ViT that used a new distillation approach to train transformers more efficiently for image classification tasks. It required less data and computing resources than the original ViT model. PvTv2 [26], which was expanded from PVT [35], proposed a flexible backbone that could achieve high output resolution for various vision tasks, particularly dense prediction tasks, while also reducing time consumption by inheriting the advantages of both CNNs and Transformers.

In addition, ref. [33] utilized a Transformer-based architecture to address the problem of detecting multi-oriented texts in images using rotated bounding boxes, but it does not work well in curved text cases. Ref. [34] proposed an end-to-end trainable framework using Transformers (DETR) to predict polygon points or Bezier control points for determining the localization of text instances. Additionally, in [36], point coordinates were directly utilized to generate position queries and progressively updated while also enhancing the spatial awareness of non-local self-attention in the Transformer. Despite significant advancements, methods utilizing the Transformer approach have still faced challenges in accurately detecting small and dense adjacent texts.

Developing robust representations is crucial for a successful scene text detector, as it necessitates the learning of discriminative features that can detect accurately text regions. As previously noted, PvTv2 [26] has demonstrated great potential as a representation of dense prediction tasks in various image applications, such as image classification, object detection, and also semantic segmentation. In this study, we introduce DenseTextPVT, which employs the PvTv2 architecture to generate improved features for dense text in scene text detection.

## 3. Methodology

### 3.1. Overall Architecture

**Figure 2:**
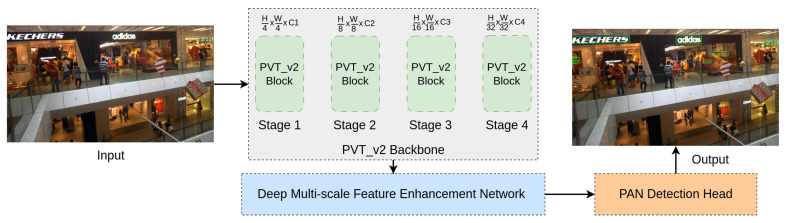
The overall framework of our DenseTextPVT approach.

**Figure 3:**
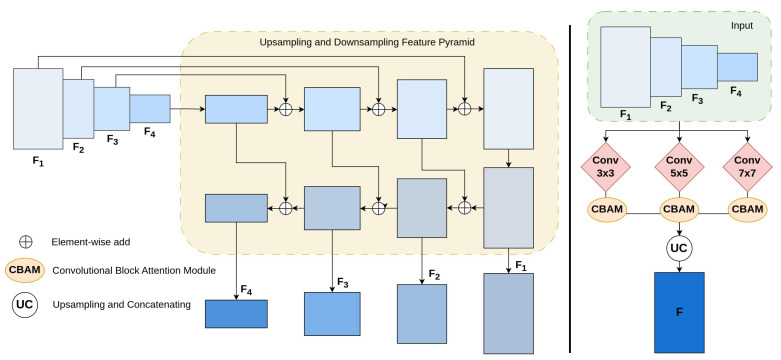
The detail of Deep Multi-scale Feature Refinement Network (DMFRN). The detail of each upsampling and downsampling feature pyramid enhancement (**left**), the overall DMFRN architecture (**right**).

**Figure 4:**
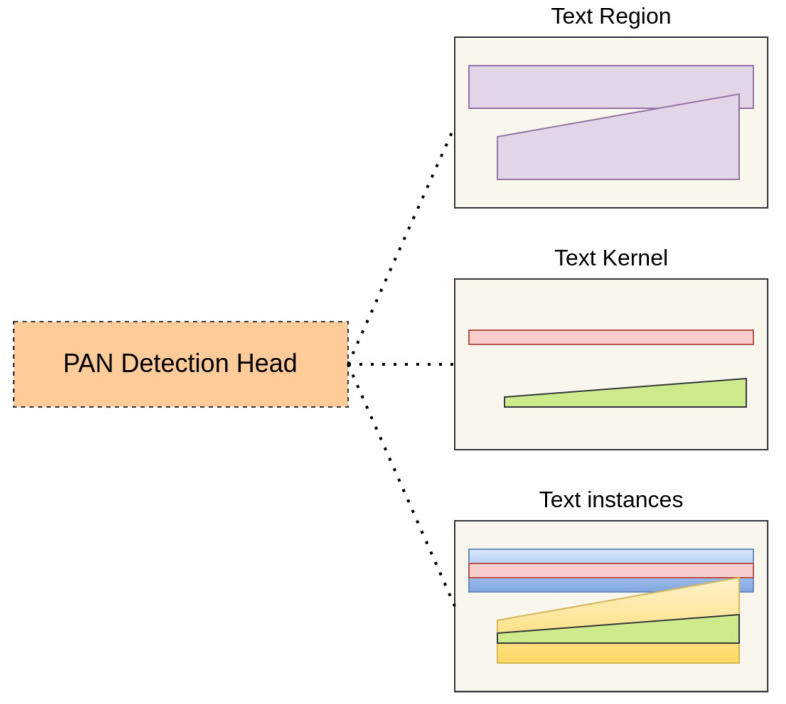
Pixel aggregation detection head.

The overall framework of our proposed method is illustrated in Figure 2. Given a scene image *I* (HxWx3), we utilized a PvTv2 backbone to extract pyramid features according to four stages, $F_{1}$, $F_{2}$, $F_{3}$, and $F_{4}$, whose strides are 4, 8, 16, and 32 pixels following the input image *I*. To refine the feature information with high resolution, we used channel attention module (CAM) and spatial attention module (SAM) approaches at $F_{1}$, $F_{2}$ and $F_{3}$, $F_{4}$ features, respectively. Then, we employed a Deep Multi-scale Feature Refinement Network (DMFRN) with three irregular kernel filters, 3×3, 5×5, and 7×7, and applied CBAM [27] at each output feature to produce multi-level features, $F_{1}^{n}$, $F_{2}^{n}$, $F_{3}^{n}$, and $F_{4}^{n}$ (n=3,5,7), with rich information on text contents of various sizes. Afterward, to prepare for the prediction stage, we scaled up $F_{2}^{n}$, $F_{3}^{n}$, and $F_{4}^{n}$ features into $F_{1}^{n}$ size and concatenated them into a single robust feature map *F*, as shown in Figure 3. Finally, our detection stage was inspired by PAN post-processing [1], which is depicted in Figure 4. In this way, our method can determine which text pixels belong to the correct text kernels, helping us accurately discriminate and mitigate the overlap phenomena between dense text regions.

### 3.2. PvTv2 Backbone

Different from convolutional neural networks such as ResNet or VGG, PvTv2 [26] serves as a versatile backbone specifically designed for various dense prediction tasks. This approach adopts the Transformer architecture and incorporates a progressive shrinking algorithm to generate feature maps of different scales using patch-embedding layers. Following the structure of [26], the algorithm consists of four pyramid stages, each comprising an overlapping patch-embedding layer and Transformer encoder layers $L_{i}$ (where *i* represents the stage of the process).

**Figure 5:**
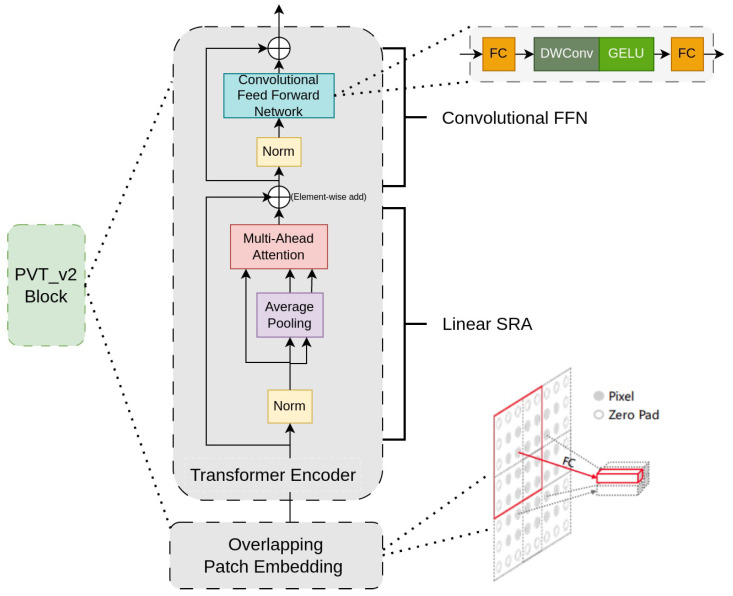
The details of PvTv2 Block. There are two main parts: overlapping patch embedding and Transformer encoder.

In each stage, the input image *I* is divided into patches of size $\frac{H}{j}\times \frac{W}{j}$ (where *j* denotes the stride sizes: 4, 8, 16, and 32 pixels), as illustrated in Figure 5. These patches are then flattened and passed through a linear projection, resulting in embedded patches of size $\frac{H}{j}\times \frac{W}{j}\times C_{i}$. PvTv2 employs an Overlapping Patch-Embedding technique by enlarging the patch window size by half of its area and utilizing convolution with zero paddings to preserve resolution. In the Transformer encoder layer, to address the computational cost associated with the attention mechanism, the authors introduced a linear shifted row attention (linearSRA) as a replacement for the traditional multi-head attention. The SRA utilizes average pooling to reduce the spatial dimensions (H,W) to a fixed size (P,P). The linearSRA can be defined as follows, with *P* set to 7:

(1) linearSRA=2×H×W×P×P×C

In addition, PvTv2 introduces a 3×3 depth-wise convolution layer with a padding size of 1 between the first fully connected (FC) and GELU layer in the feed-forward network, as shown in Figure 5. This is to eliminate the fixed-size position encoding.

The construction of feature maps with different resolutions usually loses some details of context and texture structures. To make robust our algorithm, we used channel and spatial attention modules (CAM and SAM). In general, CAM [27] captures the most meaningful and relevant information for the extracted features $F_{i}$ (*i* = 1, 2, 3, 4) through the following process: first, it performs average pooling and max pooling on the global context; next, it applies them to shared MLP; and finally, it merges feature vectors element-wise to generate a 1×1×C feature map $M_{CAM}$.

(2) $$M_{CAM}=\theta \left(MLP\left(AvgPool\left(F_{i}\right)\right)+MLP\left(MaxPool\left(F_{i}\right)\right)\right)$$

where θ represents the Sigmoid function.

Similarly, SAM [27] is also designed to extract global contextual information. It first applies average pooling and max pooling operations along the channel axis, and then it concatenates the resulting feature maps to generate a 1×H×W feature map $M_{SAM}$ using a $conv^{7\times 7}$ convolutional filter.

(3) $$M_{SAM}=\theta \left(conv^{7\times 7}\left(AvgPool\left(F_{i}\right),MaxPool\left(F_{i}\right)\right)\right)$$

### 3.3. Deep Multi-Scale Feature Refinement Network

Typically, in a pyramid structure, high-level features contain rich semantic information but lack precise location details, while low-level features have more details but are filled with background noise. Combining multi-level features can lead to better feature maps. To do that, we exploit a DMFRN with different receptive fields to detect effectively small-scale and dense adjacent texts in images. The features extracted from PvTv2, denoted as $F_{1}$, $F_{2}$, $F_{3}$, and $F_{4}$, are fed as inputs to our DMFRN stage, which consists of three convolutional kernel filters with different sizes (3×3, 5×5, and 7×7). Each block in our DMFRN stage is a U-shaped module comprising two phases: upsampling and downsampling feature pyramids, which enhances the depth of the network. By simultaneously learning the irregular kernel sizes, our model can not only enlarge receptive fields but also capture multi-level information at varying levels of text in scene images. $F_{1}^{n}$, $F_{2}^{n}$, $F_{3}^{n}$, and $F_{4}^{n}$ (*n* = 3, 5, 7) are generated by this process. Specifically, to enable the learning of relevant information in both the channel and spatial dimensions of the extracted features at each stage in the multi-scale process, we incorporate a convolutional block attention module (CBAM) [27] at each output feature, which is different from MFEN [36]. This work can boost the accuracy of the detection of dense and small text in images. Afterwards, we fuse features {$F_{1}^{3}$, $F_{2}^{3}$, $F_{3}^{3}$, $F_{4}^{3}$}, {$F_{1}^{5}$, $F_{2}^{5}$, $F_{3}^{5}$, $F_{4}^{5}$}, and {$F_{1}^{7}$, $F_{2}^{7}$, $F_{3}^{7}$, $F_{4}^{7}$} via an element-wise sum operation, respectively, to generate $F_{1}^{f}$, $F_{2}^{f}$, $F_{3}^{f}$, and $F_{4}^{f}$. Finally, we use upsampling and a concatenating algorithm to fuse these features into a final enrich feature *F*.

(4) $$F=Concate(F_{1}^{f},F_{2}^{f},F_{3}^{f},F_{4}^{f})$$

Then, we use *F* to make predictions by applying PAN [1] post-processing as in Figure 4. In this stage, we predict text instances by using similarity vectors to cluster correct text pixels with adequate text kernels.

### 3.4. Loss Function

The training loss *L* is the weighted sum of loss segmentation $L_{seg}$ and loss detection $L_{det}$. To keep the weights among these losses balanced, we set it to 0.25 experimentally.

(5) $$L=L_{seg}+0.25\times L_{det}$$

In detail, we adopt dice loss [37] to classify text/non-text in segmentation, which can be formulated as:

(6) $$L_{seg}=\frac{1}{N}\sum_{k=1}^{N}\left(1-\frac{2\times (P_{k}\cap G_{k})}{P_{k}^{2}+G_{k}^{2}}\right)$$

where *N* denotes the number of text instance samples. $P_{i}$ and $G_{i}$ represent the prediction and ground truth of the *k*th text instances. The object containing text is labeled as 1 and non-text is labeled as 0.

Additionally, $L_{det}$ represents the loss function of pixel aggregation (PA) in [1] that is applied to ensure that the text pixels are correctly associated with the appropriate text regions. This means that the distance between a text pixel and the kernel $D_{pix_{l},Ker_{l}}$ of the same *l*th text instance should be minimized.

(7) $$D_{pix_{l},Ker_{l}}=\left\{\begin{array}{cc} \leq 6, & \mathrm{if}\;pix\in (G_{l}-Ker_{l})\\ >6, & \mathrm{otherwise} \end{array}\right.$$

where $pix_{l}$ and $Ker_{l}$ define the text pixel and text kernel of *l*th text sample. $G_{l}$ is the ground truth of the *l*th text instance. The threshold of distance is set to 6 based on the PAN experiment.

## 4. Experiments and Results

### 4.1. Dataset

**TotalText** [16] comprises 1555 images, divided into 1255 training images and 300 testing images. It contains 11,459 text-bounding boxes, with 3936 and 971 instances of curved text in the training and testing sets, respectively. The number of annotated clockwise points varies for each text instance and is not fixed.

**CTW1500** [17] contains 1000 training images and 500 testing images, each with long, dense, and curved text instances. There are 10,751 text instances in total. The scenes in the dataset are challenging and diverse, and environmental factors such as blur, low resolution, and perspective distortion are present in the images.

**ICDAR 2015** [18] is a collection of incidental scene texts used in Challenge 4 on the website https://rrc.cvc.uab.es/ (accessed on 12 May 2023). The dataset contains 1000 natural images for the training process and 500 images for the testing set. It is a popular dataset for scene text detection and includes word-level text instances with multi-oriented texts, making it a useful resource for researchers in this field.

### 4.2. Implementations

During the pre-processing step, data augmentation techniques are applied for the training phase such as random crop, random rotation, random horizontal flip, and random scale, which help our model learn different scales and densities of features, leading to a better generalization ability during training and inference.

In the training phase, we only utilize the original training images of each dataset, as well as TotalText, CTW1500, and ICDAR 2015. The short side of the images is set to 640, 640, and 736 in the three datasets above, respectively. We use the PvTv2 backbone, which is a backbone for dense prediction, with strides of 4, 8, 16, and 32 pixels in input images. All the networks are optimized by the AdamW, https://pytorch.org/docs/stable/generated/torch.optim, accessed on 10 May 2023 [37] optimizer. Dice loss [38] and loss function in post-processing of PAN [1] are applied for optimization. Our model is implemented in Pytorch and trained scratch with a batch size of 4 on 1 GPU 2080Ti in 600 epochs for 150 k iterations. We use the “poly” learning rate strategy where the initial learning rate and power are set to $1\times 10^{-4}$ and 0.9, respectively.

During the inference stage, we set the batch size to 1 on 1 GPU and maintain the aspect ratio of the test images as in training phase. This ensures that the images are standardized and allows for consistent processing.

In scene text detection, regions of blurred text that are labeled as “DO NOT CARE” (###) in all datasets are commonly ignored. To address hard examples during training, online hard example mining (OHEM) [39] is utilized, with a negative–positive ratio typically set to 3. For ICDAR 2015, a minimal-area rectangle and polygon are fitted for each predicted text instance. The shrink ratio of the kernels is set to 0.7 on TotalText and CTW1500, and 0.5 on ICDAR 2015 to better fit the predicted text instance to the actual text region.

### 4.3. Evaluation Metrics

To assess the effectiveness of our proposed approach, we utilize standard metrics such as Precision (P), Recall (R), and F-measure (F). For this purpose, we consider a rectangular box containing text with a closed bounding box as True Positive (TP), while a rectangular box without any text inside is considered False Positive (FP). If there is text but no rectangular box, it is labeled as True Negative (TN), since our method failed to detect it.

In detail, Precision (P) is calculated as the ratio of the correctly identified words by our proposed method to the sum of correctly and incorrectly recognized words. It assesses the accuracy of the detected text regions. Recall (R) measures the ratio of the correct recognition to the total possible recognition at the word level. Briefly, it evaluates the ability of the method to identify all the text instances in the scene. We calculate these metrics both before and after restoration to showcase the effectiveness of our proposed approach in terms of restoring missing information, called F-measure (F). The higher the F-measure, the better the performance.

Moreover, we apply the Intersection over Union (IoU) ratio, which is used as a threshold for determining whether a predicted outcome is a True Positive (TP) or a False Positive (FP). In this paper, we set it to 0.5.

The equations of Precision, Recall, and F-measure are described below:

(8) $$P=\frac{TP}{TP+FP}$$

(9) $$R=\frac{TP}{TP+FN}$$

(10) $$F=\frac{2\times \left(P\times R\right)}{\left(P+R\right)}$$

### 4.4. Results

As presented in Table 1, Table 2 and Table 3, we compare our proposed DenseTextPVT approach with existing methods using three benchmark datasets: TotalText [16], CTW1500 [17], and ICDAR 2015 [18]. To evaluate the effectiveness of our method, we utilize the F-measure metric as in Equation (10). The results demonstrate the superior performance of our DenseTextPVT method when compared to previous algorithms.

**Figure 6:**
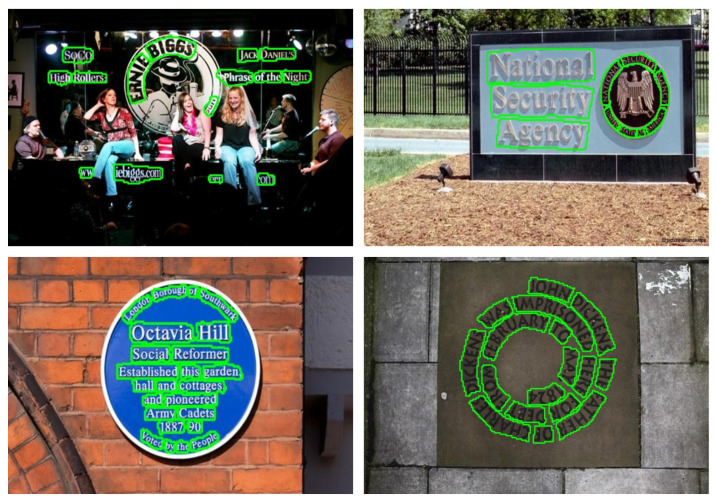
The visualization samples on TotalText [16]. It is shown that our DenseTextPVT is capable of accurately detecting dense curved texts.

Our proposed method’s effectiveness is demonstrated on the curved TotalText dataset (as shown in Table 1). Although the Recall (R) is lower compared to SegLink++ [13] and SPCNet [40], our DenseTextPVT achieves significantly higher Precision (P) and F-measure (F) scores, 89.4% and 84.7%, respectively, without relying on any external dataset. The visualization in Figure 6 clearly illustrates that our DenseTextPVT is capable of accurately detecting dense curved texts.

**Figure 7:**
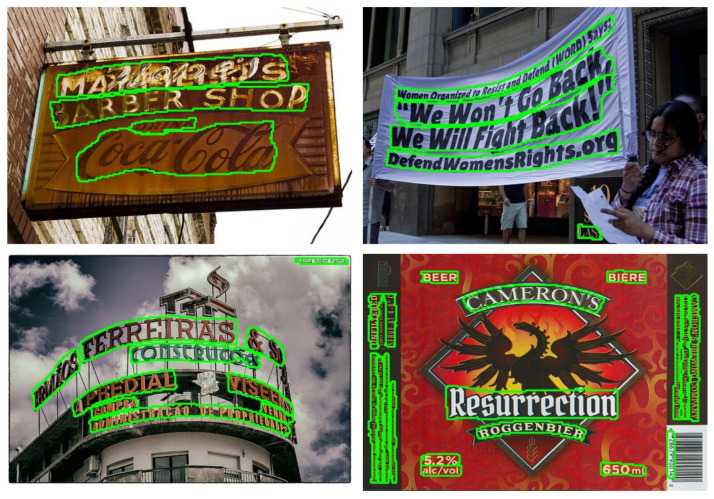
Several visualization results on long curved text lines on CTW1500 [17]. It demonstrates the accurate localization of long curved texts with dense adjacent information by our proposed method.

Similarly, our approach demonstrates strong performance on the long curved CTW1500 benchmark, achieving Precision (P) and F-measure (F) scores of 88.3% and 83.9%, respectively (as depicted in Table 2). While some algorithms, such as TextRay [30], DB [29], PAN [1], CRAFT [27], and Xiufeng et al. [32], have slightly higher Recall (R) scores, our approach outperforms the existing algorithms in terms of overall performance. Additionally, Figure 7 provides visual evidence that our proposed method accurately locates not only long curved texts but also dense adjacent text instances.

**Figure 8:**
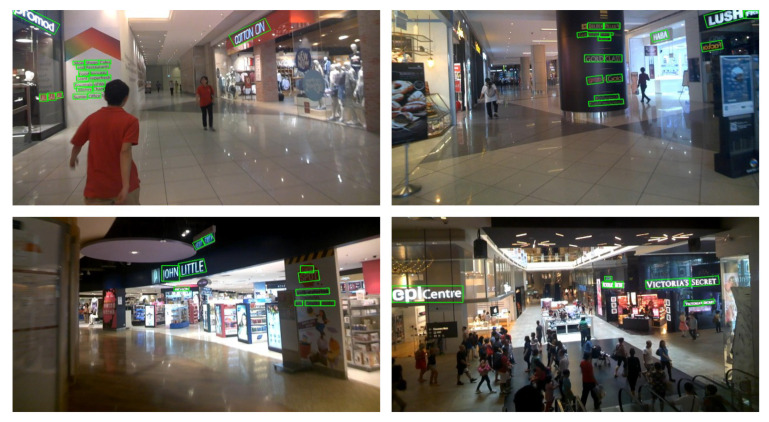
Samples demonstrate that our DenseTextPVT algorithm is capable of effectively detecting dense multi-oriented text in scene images on ICDAR 2015 [18].

When examining the results on the ICDAR 2015 dataset (as presented in Table 3), it is observed that our DenseTextPVT does not achieve the highest Precision score, such as DPTNet-Tiny [41,42] with a score of 90.3%. There is also a slight variation in the Recall score compared to algorithms like LOMO [14], MFEN [36], TextSnake [20], Xiufeng et al. [32], SegLink++ [13], and PAN [1]. However, our proposed algorithm demonstrates an impressive overall performance with an F-measure of 83.4% when trained from scratch. The visualization in Figure 8 demonstrates the effectiveness of our method in detecting dense adjacent scene texts with multiple orientations.

## 5. Conclusions

In this study, we introduced a new method, namely, DenseTextPVT, for detecting dense adjacent scene text. Our method manipulates the PvTv2 backbone with the combination of channel and spatial attention module for dense prediction, and exploits a Deep Multi-scale Feature Refinement Network to efficiently learn multi-level feature information. Afterwards, we inherit a post-processing technique in PAN to reduce overlap phenomena among text regions. Our results outperform state-of-the-art methods on several popular benchmark datasets, achieving superior F-measure scores of 84.7% on TotalText, 83.9% on CTW1500, and 83.4% on ICDAR 2015.

In the future, we plan to explore the possibility of an end-to-end framework for dense adjacent text detection. Moreover, we aim to investigate the potential of using the progressive scale expansion algorithm for segmentation mask in detection tasks, especially in benchmarks with a high density of object instances.

## Figures and Tables

**Table 1:** Quantitative detection results on TotalText. “-”/ “✓” means without/within training data. “P”, “R”, and “F” represent the Precision, Recall, and F-measure, respectively.

Method	Ext	P	R	F
EAST [8]	-	80.9	76.2	78.5
TextSnake [20]	✓	82.7	74.5	78.4
MSC [19]	✓	83.8	74.8	79.0
PSENet [7]	-	84.0	78.0	80.9
PAN [1]	-	88.0	79.4	83.5
TextRay [30]	-	83.5	77.9	80.6
SegLink++ [13]	✓	82.1	80.9	81.5
LOMO [14]	✓	87.6	79.3	83.3
SPCNet [40]	✓	83.0	82.8	82.9
PCR [10]	-	86.4	81.5	83.9
CRAFT [27]	✓	87.6	79.9	83.6
Ours_DenseTextPVT	-	89.4	80.1	84.7

**Table 2:** Quantitative detection results on CTW1500. “-”/ “✓” means without/within training data. “P”, “R”, and “F” represent the Precision, Recall, and F-measure, respectively.

Method	Ext	P	R	F
EAST [8]	-	78.7	49.1	60.4
PSENet [7]	-	80.6	75.6	78.0
PAN [1]	-	84.6	77.7	81.0
SegLink++ [13]	✓	82.8	79.8	81.3
LOMO [14]	✓	85.7	76.5	80.8
CT [12]	-	85.5	79.2	82.2
MSC [19]	✓	85.0	78.3	81.5
PCE [10]	-	85.3	79.8	82.4
TextRay [30]	-	82.8	80.4	81.6
DB [29]	✓	86.9	80.2	83.4
PAN [1]	✓	86.4	81.2	83.7
CRAFT [27]	✓	86.0	81.1	83.5
Xiufeng et al. [32]	✓	84.9	80.3	82.5
Ours_DenseTextPVT	-	88.3	79.8	83.9

**Table 3:** Quantitative detection results on ICDAR 2015. “-”/ “✓” means without/within training data. “P”, “R”, and “F” represent the Precision, Recall, and F-measure, respectively.

Method	Ext	P	R	F
EAST [8]	-	83.6	73.5	78.2
PSENet [7]	-	81.5	79.7	80.6
DPTNet-Tiny [41]	✓	90.3	77.4	83.3
LOMO [14]	✓	83.7	80.3	82.0
TextSnake [20]	✓	84.9	80.4	82.6
Xiufeng et al. [32]	-	85.8	79.7	82.6
MFEN [38]	-	84.5	79.7	82.0
SegLink++ [13]	✓	83.7	80.3	82.0
MSC [19]	✓	86.6	78.4	82.3
PAN [1]	-	82.9	77.8	80.3
PAN [1]	✓	84.0	81.9	82.9
Ours_DenseTextPVT	-	87.8	79.4	83.4

## Data Availability

Not applicable.

## Abbreviations

The following abbreviations are used in this manuscript:

PvTv2	Pyramid Vision Transformer
CAM	Channel Attention Module
SAM	Spatial Attention Module
CBAM	Convolutional Block Attention
DNNs	Deep Neural Networks
DMFRN	Deep Multi-scale Feature Refinement Network
PA	Pixel Aggregation
PAN	Pixel Aggregation Network
LinearSRA	Linear Shifted Row Attention
FC	Fully Connected
FFN	Feed Forward Network
P	Precision
R	Recall
F	F-measure

## References

[1] Wang W., Xie E., Song X., Zang Y., Wang W., Lu T., Yu G., Shen C. Efficient and accurate arbitrary-shaped text detection with pixel aggregation network. Proceedings of the IEEE/CVF International Conference on Computer Vision.

[2] Liao M., Wan Z., Yao C., Chen K., Bai X. (2022). Real-time scene text detection with differentiable binarization and adaptive scale fusion. IEEE Trans. Pattern Anal. Mach. Intell..

[3] Zhang S.X., Zhu X., Chen L., Hou J.B., Yin X.C. (2022). Arbitrary Shape Text Detection via Segmentation with Probability Map. IEEE Trans. Pattern Anal. Mach. Intell..

[4] Tang J., Zhang W., Liu H., Yang M., Jiang B., Hu G., Bai X. Few Could Be Better Than All: Feature Sampling and Grouping for Scene Text Detection. Proceedings of the IEEE/CVF Conference on Computer Vision and Pattern Recognition.

[5] Yin X.-C., Yin X., Huang K., Hao H.-W. (2013). Robust text detection in natural scene images. IEEE Trans. Pattern Anal. Mach. Intell..

[6] Chen Z., Wang J., Wang W., Chen G., Xie E., Luo P., Lu T. (2021). FAST: Searching for a Faster Arbitrarily-Shaped Text Detector with Minimalist Kernel Representation. arXiv.

[7] Wang W., Xie E., Li X., Hou W., Lu T., Yu G., Shao S. Shape robust text detection with progressive scale expansion network. Proceedings of the IEEE/CVF Conference on Computer Vision and Pattern Recognition.

[8] Zhou X., Yao C., Wen H., Wang Y., Zhou S., He W., Liang J. East: An efficient and accurate scene text detector. Proceedings of the IEEE Conference on Computer Vision and Pattern Recognition.

[9] Zhou B., Khosla A., Lapedriza A., Oliva A., Torralba A. Learning deep features for discriminative localization. Proceedings of the IEEE Conference on Computer Vision and Pattern Recognition.

[10] Dai P., Zhang S., Zhang H., Cao X. Progressive contour regression for arbitrary-shape scene text detection. Proceedings of the IEEE/CVF Conference on Computer Vision and Pattern Recognition.

[11] Baek Y., Lee B., Han D., Yun S., Lee H. Character region awareness for text detection. Proceedings of the IEEE/CVF Conference on Computer Vision and Pattern Recognition.

[12] Sheng T., Chen J., Lian Z. (2021). Centripetaltext: An efficient text instance representation for scene text detection. Adv. Neural Inf. Process. Syst..

[13] Shi B., Xiang B., Serge B. Detecting oriented text in natural images by linking segments. Proceedings of the IEEE Conference on Computer Vision and Pattern Recognition.

[14] Zhang C., Borong L., Zuming H., Mengyi E., Junyu H., Errui D., Xinghao D. Look more than once: An accurate detector for text of arbitrary shapes. Proceedings of the IEEE/CVF Conference on Computer Vision and Pattern Recognition.

[15] He W., Zhang X.-Y., Yin F., Liu C.-L. Deep direct regression for multi-oriented scene text detection. Proceedings of the 2017 IEEE International Conference on Computer Vision (ICCV).

[16] Kheng C.C., Chan C.S. (2017). TotalText: A comprehensive dataset for scene text detection and recognition. Proceedings of the 2017 14th IAPR International Conference on Document Analysis and Recognition (ICDAR), Kyoto, Japan, 9–15 November 2017.

[17] Liu Y., Jin L., Zhang S., Zhang S. (2017). Detecting curve text in the wild: New dataset and new solution. arXiv.

[18] Karatzas D., Gomez-Bigorda L., Nicolaou A., Ghosh S., Bagdanov A., Iwamura M., Matas J., Neumann L., Chandrasekhar V.R., Lu S. ICDAR 2015 competition on robust reading. Proceedings of the 2015 13th International Conference on Document Analysis and Recognition (ICDAR).

[19] Xue C., Shijian L., Wei Z. (2019). MSR: Multi-scale shape regression for scene text detection. arXiv.

[20] Long S., Jiaqiang R., Wenjie Z., Xin H., Wenhao W., Cong Y. Textsnake: A flexible representation for detecting text of arbitrary shapes. Proceedings of the European Conference on Computer Vision (ECCV).

[21] Vaswani A., Shazeer N., Parmar N., Uszkoreit J., Jones L., Gomez A.N., Kaiser Ł., Polosukhin I. (2017). Attention is all you need. Adv. Neural Inf. Process. Syst..

[22] Carion N., Massa F., Synnaeve G., Usunier N., Kirillov A., Zagoruyko S. (2020). End-to-end object detection with transformers. Computer Vision–ECCV 2020: 16th European Conference, Glasgow, UK, 23–28 August 2020.

[23] Ze L., Lin Y., Cao Y., Hu H., Wei Y., Zhang Z., Lin S., Guo B. Swin transformer: Hierarchical vision transformer using shifted windows. Proceedings of the IEEE/CVF Conference on Computer Vision and Pattern Recognition.

[24] Dosovitskiy A., Beyer L., Kolesnikov A., Weissenborn D., Zhai X., Unterthiner T., Dehghani M., Minderer M., Heigold G., Gelly S. An image is worth 16 × 16 words: Transformers for image recognition at scale. Proceedings of the International Conference on Learning Representations.

[25] Hugo T., Cord M., Douze M., Massa F., Sablayrolles A., Jégou H. (2021). Training data-efficient image transformers & distillation through attention. Int. Conf. Mach. Learn..

[26] Wang W., Xie E., Li X., Fan D., Song K., Liang D., Lu T., Luo P., Shao L. (2022). Pvt v2: Improved baselines with pyramid vision transformer. Comput. Vis. Media.

[27] Woo S., Park J., Lee J.-Y., Kweon I.S. Cbam: Convolutional block attention module. Proceedings of the European Conference on Computer Vision (ECCV).

[28] Zhu Y., Chen J., Liang L., Kuang Z., Jin L., Zhang W. Fourier contour embedding for arbitrary-shaped text detection. Proceedings of the IEEE/CVF Conference on Computer Vision and Pattern Recognition.

[29] Liao M., Wan Z., Yao C., Chen K., Bai X. Real-time scene text detection with differentiable binarization. Proceedings of the AAAI Conference on Artificial Intelligence.

[30] Wang F., Chen Y., Wu F., Li X. Textray: Contour-based geometric modeling for arbitrary-shaped scene text detection. Proceedings of the 28th ACM International Conference on Multimedia.

[31] Dang Q.-V., Lee G.-S. (2021). Document image binarization with stroke boundary feature guided network. IEEE Access.

[32] Jiang X., Xu S., Zhang S., Cao S. (2020). Arbitrary-shaped text detection with adaptive text region representation. IEEE Access.

[33] Zobeir R., Naiel M.A., Younes G., Wardell S., Zelek J.S. Transformer-based text detection in the wild. Proceedings of the IEEE/CVF Conference on Computer Vision and Pattern Recognition.

[34] Zobeir R., Younes G., Zelek J. Arbitrary shape text detection using transformers. Proceedings of the 2022 26th International Conference on Pattern Recognition (ICPR).

[35] Wang W., Xie E., Li X., Fan D., Song K., Liang D., Lu T., Luo P., Shao L. Pyramid vision transformer: A versatile backbone for dense prediction without convolutions. Proceedings of the IEEE/CVF Conference on Computer Vision and Pattern Recognition.

[36] Dinh M.-T., Lee G.-S. Arbitrary-shaped Scene Text Detection based on Multi-scale Feature Enhancement Network. Proceedings of the Korean Information Science Society Conference.

[37] Sudre C.H., Li W., Vercauteren T., Ourselin S., Cardoso M.J. (2017). Generalised dice overlap as a deep learning loss function for highly unbalanced segmentations. Deep Learning in Medical Image Analysis and Multimodal Learning for Clinical Decision Support: Third International Workshop, DLMIA 2017, and 7th International Workshop, ML-CDS 2017, Held in Conjunction with MICCAI 2017, Québec City, QC, Canada, 14 September 2017.

[38] Loshchilov I., Hutter F. (2017). Decoupled weight decay regularization. arXiv.

[39] Shrivastava A., Gupta A., Girshick R. Training region-based object detectors with online hard example mining. Proceedings of the IEEE Conference on Computer Vision and Pattern Recognition.

[40] Enze X., Zang Y., Shao S., Yu G., Yao C., Li G. Scene text detection with supervised pyramid context network. Proceedings of the AAAI Conference on Artificial Intelligence.

[41] Lin J., Jiang J., Yan Y., Guo C., Wang H., Liu W., Wang H. (2022). DPTNet: A Dual-Path Transformer Architecture for Scene Text Detection. arXiv.

[42] Deng D., Liu H., Li X., Cai D. Pixellink: Detecting scene text via instance segmentation. Proceedings of the AAAI Conference on Artificial Intelligence.

